# Gluco-regulation & type 2 diabetes: entrenched misconceptions updated to new governing principles for gold standard management

**DOI:** 10.3389/fendo.2024.1394805

**Published:** 2024-06-12

**Authors:** Stanley S. Schwartz, Mary E. Herman

**Affiliations:** ^1^ Main Line Health, Wynnewood, PA, and University of Pennsylvania, Philadelphia, PA, United States; ^2^ Social Alchemy: Building Physician Competency Across the Globe, Sacatepéquez, Guatemala

**Keywords:** type 2 diabetes, GLP-1 receptor agonists, SGLT-2 inhibitors, beta cells, type 2 diabetes remission, cardiorenal axis, kidney disease, type 2 diabetes pharmacotherapy

## Abstract

Our understanding of type 2 diabetes (T2D) has evolved dramatically. Advances have upended entrenched dogmas pertaining to the onset and progression of T2D, beliefs that have prevailed from the early era of diabetes research—and continue to populate our medical textbooks and continuing medical education materials. This review article highlights key insights that lend new governing principles for gold standard management of T2D. From the historical context upon which old beliefs arose to new findings, this article outlines evidence and perspectives on beta cell function, the underlying defects in glucoregulation, the remediable nature of T2D, and, the rationale supporting the shift to complication-centric prescribing. Practical approaches translate this rectified understanding of T2D into strategies that fill gaps in current management practices of prediabetes through late type 2 diabetes.

## Highlights

- The early research era of diabetes planted several incorrect notions about the disease state, assumptions that are contrary to rational approaches to its clinical management. Entrenched misconceptions must be revisited and rectified.
- Prediabetes and early T2D are not necessarily intractable (“inexorable”) states widely described. Beta cells are resilient and can be harnessed to restore glucoregulation and euglycemia in many patients. This restoration is, in fact, already routinely accomplished through diet and lifestyle modifications.
- The first objective should be to re-enlist the inherent resilience of beta cells to remit dysglycemia. When diet and lifestyle modifications are not adequate, established short-term intensive pharmacotherapy regimens are available and can be employed.
- Hyperglycemia in T2D is not a single disease across all patients; it does not arise from the same origins but rather through a variety of derangements contributing to the hyperglycemia. An individualized therapy approach targets each driver of hyperglycemia at work. This is a superior approach to sequential add-on therapy.
- Cognitive dysfunction and dementia-related disorders, including Alzheimer’s disease, are among an expanded list of diabetes-related long-term outcomes. Intensive glucose control is imperative for these long-term complications as well as ‘classic’ long-term outcomes.
- Glucose variability is associated with poorer outcomes. A chief goal of therapy is to lower plasma glucose levels while avoiding severe hypoglycemic episodes. Of equal concern are mild, often ‘silent’ forms of hypoglycemia, which have been underappreciated to date.
- As much as 40% of hyperglycemia is estimated to be due to genetic and other factors—variables that may not be able to be countered through antidiabetes approaches.
- Sulfonylureas and human insulin present poorer benefit–risk profiles than other available agents. Each of these classes are well documented for weight gain, susceptibility to hypoglycemia, and cardiovascular risks.
- The pleiotropic effects of antidiabetic agents have proven important for both short- and long-term patient outcomes. When considering individual agents, the pleiotropic benefits are increasing priorities in treatment of choice.

## Type 2 diabetes: foundations, fallacies, foibles

Research has greatly advanced our understanding of the pathophysiology of T2D. Contemporary tools of research are light years ahead of those that informed our early assumptions about dysglycemia. Many of these outdated notions were laid down decades ago as the basic tenets of the disease, but continue to populate medical textbooks today. Today’s T2D is, proverbially, ‘not your mother’s T2D’.

To some extent, the early basis for T2D was derived in the mid-20^th^ century based on what is ‘wasn’t’ – namely, how it’s presentation was presumed to be distinct from type 1 diabetes (T1D). Unlike the destruction of beta cells in T1D, T2D was characterized as a disease of lifestyle choice. [This was only partially accurate, as it has been estimated that as much as 40% of the factors contributing to the development of T2D are not readily modifiable by diet and lifestyle modification (1–3)]. It is now understood that T1D and T2D share a substantial overlap of etiological factors. For example, similar to T1D, T2D has autoimmune and genetic components, albeit different from those associated with T1D.

As an expanding antidiabetes armamentarium makes prescribing more complex, an accurate understanding of the underpinnings of the disease is essential for a rational approach to its management. The *2024 American Diabetes Association Standards of Care* encapsulates best practices, but at ~300 pages (4–9), it is voluminous. Those guideline crafters agree, *“The management of hyperglycaemia in type 2 diabetes has become extraordinarily complex with the number of glucose-lowering medications now available. Patient-centred decision making and support and consistent efforts to improve diet and exercise remain the foundation of all glycaemic management. Initial use of metformin, followed by addition of glucose-lowering medications based on patient comorbidities and concerns is recommended as we await answers to the many questions that remain.”* (9)

This article summarizes key advances that redefine and rectify our understanding of T2D. It translates this knowledge into practical approaches for the clinic.

**Table 1** details critical updates about T2D that we shortlisted for readers. The evidence of the updated nature, progression, and malleability of the beta cell and glucoregulatory apparatus is elaborated throughout this paper.

**Table 1:** Selected Fallacies, Foibles, and Rectified New Foundations.

Fallacious or Outmoded Presumptions	Updated/Corrected Concepts	Selected References
Etiology		
Overweight and obesity is the chief cause of T2D	Excess nutrient intake is a greater contributor to development of T2D than BMI. BMI measurements may confounds diagnosis and management, as these do not estimate visceral fat, the predictor of T2D.	ReTune Study. Taylor 2023 (10) Shuster et al., 2012 (11)
T1D is an autoimmune disorder; T2D is a condition of poor lifestyle	Similar to T1D, T2D has autoimmune and genetic components - albeit different ones than those associated with T1D. Environmental influences, such as gut biome, impact development of T2D. T2D is also influenced by numerous other metabolic factors.	Schwartz et al. DiaCare 2016 (12); Schwartz et al., 2017 (13); Hoffman et al., 2021 (2); Letchumanan et al., 2022 (1); Fang et al., 2023 (3); Handelsman et al., 2023 (14)
If A1c levels are not attained goals with attentive medical management, the patient is nonadherent	Lack of attainment of A1c targets may not be due to treatment failure or lack of adherence. An estimated 40% of hyperglycemia is due to genetic or other factors that may be refractory to glucose-lowering therapies.	Hoffman et al., 2021 (2); Letchumanan et al., 2022 (1); Fang et al., 2023 (3)
Natural History/Progression		
T2D is an irreversible condition marked by progressive, “inexorable” march of T2D	Beta cells possess an inherent resilience in early and prediabetes, and, likely, until the beta cells completely ‘peter out’ late in the course of the disease. Beta cells may enlisted back into service by easing the nutrient overload or insulin resistance that is taxing beta cells, as example.	Schwartz et al. DiaCare 2016 (12); Schwartz et al., 2017 (13); Retnakaran et al., 2023 (15)
*Ominous Octet* encapsulates the defects leading to hyperglycemia	Updated to *‘Egregious Eleven’*, at least eleven distinct defects contributing to hyperglycemia are known. Of translational import, identification of defects at work in individual patients guide choice of therapy.	Schwartz et al. DiaCare 2016 (12); Schwartz et al., 2017 (13)
Remission of T2D is not mandated by 2023 guidelines, and, therefore, should not be considered	In 2023, an expert panel clearly outlined that, while certain aspects of T2D remission still need to be ironed out, physicians are encouraged to prioritize remission as a goal of therapy, using their own discretion as to candidate patients.	Riddle et al., 2023 (16)
A1c Targets		
Intensive glucose control is ‘hard won’ in real-world care	Attainment of A1c targets (7.0%) is realizable in most patients, using regimens comprised of newer agents. Precision medicine can be practiced by targeting the particular drivers of hyperglycemia present in a given patient. This limits use of ineffective agents within regimens.	Schwartz et al. DiaCare 2016 (12); Schwartz et al., 2017 (13); Riddle 2018 (17); Berkovic et al., 2020 (18); Davies et al., 2022 (19)
Hyperglycemia is the chief concern in T2D	Glucose variability – fluctuations between hyperglycemia or hypoglycemia – is as consequential to managing dysglycemia as is hyperglycemia.	Umpierrez et al., 2018 (20); Ceriello et al., 2019 (21); Martinez et al., 2021 (22); Riddle et al., 2018 (17)
Hypoglycemia is the rate-limiter of tight, intensive treatment	Reduced reliance on sulfonylureas or human insulin, and preferential use of alternate approaches and pharmacotherapies, can successfully avoid much of the hypoglycemia with antidiabetes management in the past.	Schwartz et al. DiaCare 2016 (12); Schwartz et al., 2017 (13); Herman et al., 2017 (23); Riddle 2018 (17); Davies et al., 2022 (19); Azouley et al., 2017 (24)
Complications & Outcomes		
Problems associated with hypoglycemia arise from severe hypoglycemic episodes	Nonsevere hypoglycemic episodes are as detrimental as severe hypoglycemia episodes’. ‘Silent’ hypoglycemia can occur frequently, and, without awareness by patients. Nonsevere hypoglycemic episodes are associated with development of long-term complications, such as dementia-related disorders.	Lin et al., 2013 (25); Seaquist et al., 2013 (26); Lin et al., 2020 (27)
The long-term complications of diabetes include: cardiovascular disease, peripheral artery disease, nephropathy, retinopathy, and neuropathy	The roster of long-term complications of diabetes has expanded to include gastrointestinal problems, stroke, dental disease, immunocompromise, cancer, cognitive decline, and onset of dementia-related disorders (recently termed ‘type 3 diabetes’).	Schwartz et al., 2017 (13); Antar et al., 2023 (28); Banks et al., 2012 (29)
Diabetes-related CV and renal complications are not mitigable	Risk of complications within the cardiorenal axis is mitigable by choice of antidiabetes classes. Sulfonylureas and human insulin have been shown to have greater associations with these long-term outcomes, whereas GLP-1 receptor agonists and SGLT-2 inhibitors have evidence for slowing or preventing CV or renal disease. Accordingly, the latter two have been recommended as treatments of choice for patients with, or at risk, of these complications.	Ke et al., 2021 (30); Evans et al., 2023 (31); Mannucci et al., 2020 (32); Schwartz et al, Postgraduate Medicine, 2016 (33); Herman et al., 2017 (23)
Treatments		
Choice of treatment is guided by extent of glucose-lowering needed	‘C*omplication-centric prescribing’* is dictated by AACE as well as ADA guidelines. It allows tailored therapy, as well as capitalizes on pleiotropic actions of specific classes that help ameliorate complications.	Samson et al., 2023 (34); Elsayed et al., 2024 (4, 5, 7, 8, 35)
In face of treatment failure, add-on therapy should be initiated	At least eleven drivers of hyperglycemia are known to exist. Treatment should, whenever possible, target the drivers of hyperglycemia at work in a given patient. The corollary of this is that agents that are ineffective may not align with a driver, and should be discontinued to reduce polypharmacy.	Schwartz et al. DiaCare 2016 (12); Schwartz et al., 2017 (13)
Approved doses are the generally the ideal dosages	The lowest dose that ‘gets the job done’ is the ideal dosage for a given patient. Likewise, the least number of agents to reach A1c targets should be used.	Holford et al., 2022 (36); Schwartz et al. DiaCare 2016 (12); Schwartz et al., 2017 (13)

## Upending the belief in a fatalistic “inexorable” march of T2D

A major change that at the foundational level of our understanding of T2D is the dismantlement of the assumed ‘inexorable march of T2D’. This presumption arose in the early era of T2D, when the diagnostics at the time identified T2D in its advanced stages - at a juncture when the beta cells had exhausted as much as 90% of their capacity and their function could not be salvaged. Today’s diagnostics, however, detect T2D early in the course of the disease, as early as prediabetes, when the beta cells are still robust and resilient. A second constraint in the early era of diabetes management was treatment options limited to ‘oafish’ drugs such as sulfonylureas. Sulfonylureas have long been speculated to overextend beta cells, pushing the cells to exhaustion. This remains a contentious proposition, but is nonetheless supported by strong associations of use of sulfonylureas with rapid treatment failure (37) accompanied by loss of beta cell mass (38–40), and, in preclinical studies, evidence of sulfonylurea-induced beta cell apoptosis (41).

Today’s first-line standard of care is diet and lifestyle modifications to remit hyperglycemia. Physicians routinely ‘turn back’ dysglycemia in their routine clinical practice through these relatively simple changes (42–46). Once pharmacotherapy is initiated, however, standard of care is built on the assumption the that the course of T2D becomes irrevocably unidirectional, towards loss of endogenous glucose control. This overlooks the inherent resilience of beta cells and the glucoregulatory apparatus to restore normoglycemia when aided. It obviates a new gold standard that dysglycemia may be mitigable – even if first efforts at diet and lifestyle modification failed.

Beta cells do indeed ‘peter out’, but this typically occurs in the advanced stages of the disease. Before this advent, the rational approach to optimal care is to ‘reset’ metabolism in an attempt to let the beta cells and glucoregulatory apparatus restore gluco-homeostasis.

There are ample examples of remission in real-world practice. Gestational diabetes and glucocorticoid-induced diabetes are associated with transient hyperglycemia, from which normoglycemia can be restored, that is, the glucoregulatory apparatus given the support to ‘right’ itself. The COVID-19 pandemic brought a record number of patients into the clinic with hyperglycemia—individuals who had been normoglycemic prior to the pandemic stay-at-home orders. These cases of new-onset hyperglycemia present in conjunction with healthy beta cells, which are readily remediable in most patients.

Given this reevaluation of T2D as ‘reversible’ rather than ‘inexorable’ (at least until the late stage of the disease) (14, 47), the primary objectives of disease management are first to remit hyperglycemia and, second, to prioritize the preservation of beta cell function, the common denominator upon which gluco-stasis relies (14, 47, 48).

## The plasticity of beta cells

It has long been understood that prenatal exposure to maternal conditioning influences the risk for obesity and T2D later in life (*reviewed by* these authors [SSS] in Amrom & Schwartz 2022 (49). The subsequent development of genetic tools led to the discovery that epigenetic programming is behind this phenomenon, allowing for phenotypic plasticity and adaptation (50). This constitutes an evolutionary strategy to adapt to highly variable environmental conditions and nutrient availability. (In the modern day, this evolvability will prove detrimental in the face of affluence-related overnutrition).

Beta cells are endowed with the remarkable capacity to dedifferentiate, transdifferentiate and redifferentiate. In response to changing environmental or nutrient conditions, metabolic stress, or sustained hyperglycemia. Beta cells can be programmed to dedifferentiate into immature cells, then transdifferentiate these immature cells into pancreatic alpha cells or other islet cell types, as needed by the organism (50–52). It is imperative to supplant old tenets of the unyielding nature of the natural history of T2D (14, 33, 47, 48). This plasticity supports the idea that sustained hyperglycemia can be reversed.

## Is it possible to enlist beta cells back into service?

Remission is defined as an HbA1c <6.5% (48 mmol/mol) measured at least 3 months after cessation of glucose-lowering pharmacotherapy (16). As discussed, reduced nutrient intake is one means to normalize the glucoregulation. This dietary changes eases the work placed on the beta cells and recalibrates insulin resistance, allowing the glucoregulatory apparatus to ‘right’ itself.

The phenomenon of remission of hyperglycemia has been most dramatically demonstrated following bariatric surgery (53–55). Bariatric surgery has been renamed ‘metabolic surgery’ for its efficacy in reversing dysmetabolism (54–56).

Importantly, remission can be achieved in general practice with the use of pharmacotherapy. Short-term, intensive pharmacotherapy has been shown to restore euglycemia in clinical studies (57–59). In our practice (SSS), remission has been achieved through short-term intensive pharmacotherapy even in patients presenting A1c values as high as 9% at the start of intervention. In one clinical “induction” study, short-term intensive insulin therapy followed by “maintenance” with metformin restabilized glucose over a course of two years in nearly half of the patients studied. In some patients, remission persisted after the withdrawal of glucose-lowering pharmacotherapy, supporting the idea that short-term support could ‘reset’ the glucoregulatory apparatus. The finding that improvements in beta cell function preceded improvements in hepatic insulin sensitivity in the trial setting suggests a central role for the beta cells restoring overall gluco-stasis (60).

Remission of T2D, however, should not be misconstrued as a ‘cure’. A proclivity toward hyperglycemia may remain, depending on the drivers contributing to the patient’s dysglycemia, and, patient adherence. It is also important to understand that, despite mitigation of hyperglycemia, underlying abnormalities may persist. Irreversible damage from glucolipotoxicity, which manifests as a long-term complication of diabetes, may not be remittable or reversible (61). Accordingly, screening for diabetes-related complications should be ongoing, including in patients who reestablish normoglycemia. In patients with cardiorenal compromise or who are at risk of compromise, GLP-1 receptor agonists and SGLT-2 inhibitors have each been shown to provide protection. The relative benefits of this protective effect of these treatments must be weighed against the delay of their use in patients in T2D remission.

In 2023, an expert panel clearly outlined that, while aspects of T2D remission, such as the above example, still need to be ironed out, physicians are encouraged to proceed to initiate remission as a goal of therapy, using their own discretion as to candidate patients (16).

Expended efforts to remit T2D represents a major conceptual and clinical leap in the routine management of the disease. By enlisting the body’s glucoregulatory apparatus to resume euglycemia, these authors envision a new standard for care in which T2D can potentially be averted for a decade or more in a large subset of patients.

## From the *Ominous Octet* to the *Egregious Eleven*

Dysglycemia is accompanied by derangements in its three key defects: beta cell dysfunction, insulin resistance, and hyperinsulinemia. These three variables become head-locked, each of which potentiates dysfunction of the others in a vicious cycle. The composite of defects contributing to hyperglycemia is widely known as the *Ominous Octet* (62).

**Figure 1:**
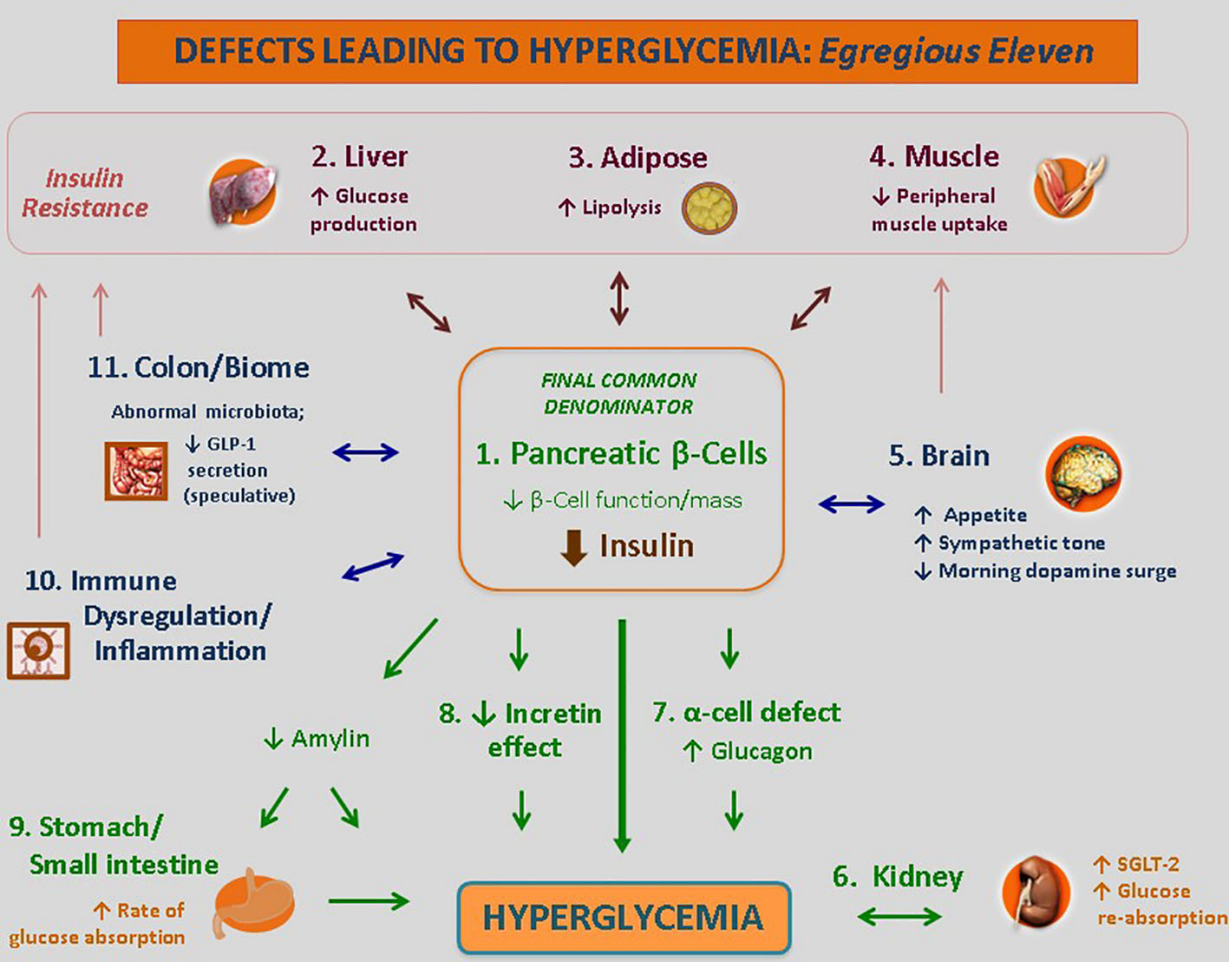
The use of the Egregious Eleven as a guide for patient-centric therapy. Targeted therapies for each of the current pathways mediating hyperglycemia, weight reduction and CV benefits based on The Beta-Cell–Centric Model. GLP-1, glucagon-like peptide 1; QR, quick release. † - Weight reducing agent. * - Potential CV benefit shown for at least one member of the class. Adapted from ‘The Time Is Right for a New Classification System for Diabetes: Rationale and Implications of the Beta -Cell–Centric Classification Schema’ appeared in Schwartz et al, DiaCare, 2016 (12). Reuse permission granted.

Current research allows the core defects of T2D to be expanded to the *Egregious Eleven*, as described by these authors. These acknowledge the influence of the stomach and small intestine, immune dysregulation, and the constitution of the gut microbiome on glucose regulation (12) (**Figure 1**). Additional defects are emerging. Neurotransmitters such as dopamine and hormones such as testosterone each possesses gluco-modulatory effects (63, 64). In cross talk, the renin–angiotensin system–insulin signaling pathway and vitamin D have also been shown modulate insulin secretion (65).

Another leap in understanding is that A1c levels are not the sole target or consideration for choice of therapy. The various defects at work as the drivers of hyperglycemia in a given patient varies from case to case but is rarely limited to only one pathway. Moreover, within a given patient, these defects may vary across disease duration.

**Figure 2:**
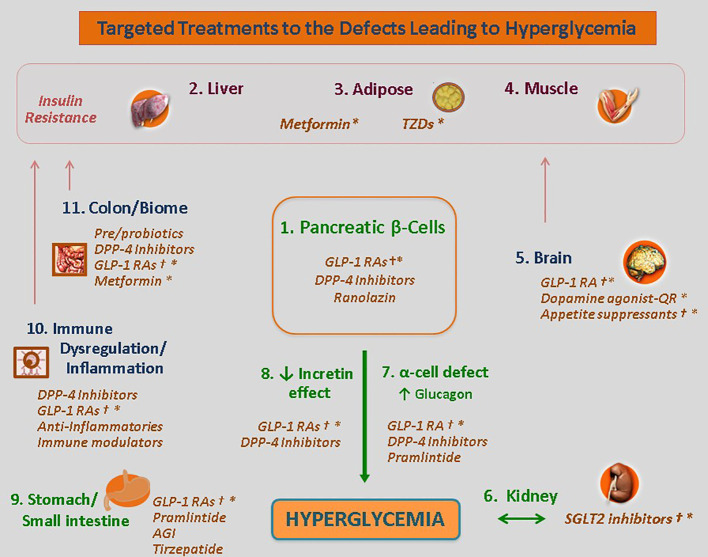
Targeted Treatments for the Defects Leading to Hyperglycemia. The use of the Egregious Eleven as a guide for patient-centric therapy. Targeted therapies for each of the current pathways mediating hyperglycemia, weight reduction and CV benefits based on the beta-cell–centric model. GLP-1, glucagon-like peptide 1; QR, quick release. † - Weight reducing agent. * - Potential CV benefit shown for at least one member of the class. Adapted from ‘The Time Is Right for a New Classification System for Diabetes: Rationale and Implications of the Beta-Cell-Centric Classification Schema’, which appeared in Schwartz et al., DiaCare, 2016 (12). Reuse permission granted.

It follows that individualized treatment (‘precision medicine’) can be realized through targeted therapies aimed at the specific mediating pathways driving hyperglycemia in the given patient. Some defects can be readily identified; other defects may be assessed through the effectiveness, or lack thereof, an antidiabetes class in that patient (**Figure 2**).

The lowest dose that ‘gets the job done’ is the ideal dosage for a given patient. Likewise, the least number of agents to reach A1c targets should be used (12, 13, 23, 36, 66). This finding argues against prescribing habits that ‘blindly’ resort to add-on therapy in the face of treatment failure. The customization of treatments aimed at mitigating as many drivers of hyperglycemia in a given patient as possible is optimal. There is no rationale to use any drug class that is not effective or aligned with one of a driver present in the given patient.

## Long-term outcomes of sustained dysglycemia: the full complement of complications

Our large-scale studies, together with the mining of patient registry databases, have been successful in resolving the full complement of T2D-related long-term complications and has expanded the roster of diabetes-related long-term complications beyond the classic complications (cardiovascular disease, peripheral artery disease, nephropathy, retinopathy, and neuropathy). In addition to the five outcomes usually considered in the clinic, stroke, gastrointestinal problems, dental disease, immunocompromise, cognitive dysfunction, and even cancer are now understood to be deleteriously affected by sustained hyperglycemia [*reviewed by* these authors (SSS, MEH) in Schwartz et al., 2017 (13)].

The clear association between sustained hyperglycemia and neuronal impairment and cognitive decline is particularly noteworthy (29, 67, 68). Diabetes predisposes individuals not only to cognitive decline but also to the onset of dementia-related disorders, including Alzheimer’s disease (69, 70). Cognition has been shown to be a modifiable target for clinical management (71, 72), and argues for intensive glycemic control as an imperative in the long-term management of T2D and overall wellness of the patient.

## ‘Complication-centric prescribing’

The *ADA Standards of Care in Diabetes - 2024* detail approaches to managing comorbid conditions in patients with T2D (4–9). This is anatomized as ‘complication-centric prescribing’ according to the *American Diabetes Association Standards of Care in Diabetes—2024* (9) and is largely consistent with the recommendations of the *American Association of Clinical Endocrinology* (34). A windfall of large-scale trials that evaluated newer antidiabetic agents was the finding that certain classes possess pleiotropic benefits—benefits distinct from the glucose-lowering action of the drug. As many of these pleiotropic benefits have been shown to reduce one or more long-term outcomes, this provides a new level of individualized care for patients at risk.

Now mandated in the treatment guideline, complication-centric prescribing moves away from indiscriminate treatment of choice from a ‘laundry list’ of agents. Instead, it supports tailoring the choice of therapy for its therapeutic benefit to specific comorbid conditions. The evidence from large-scale trials that GLP-1 receptor agonists and SGLT-2 inhibitors slow or prevent CV or renal disease, is chief example. Analyses are underway to resolve individual treatments for their impact on other long-term complications, including cognition, a major consideration for quality of life, overall wellness, and chronic disease management in older individuals.

## Insulin therapy revisited: benefit:risk considerations

In addition to pleotropic benefits, antidiabetes agents may also possess deleterious off-target effects. One recently released guideline (*Obesity, diabetes mellitus, and cardiometabolic risk: An Obesity Medicine Association (OMA) Clinical Practice Statement (CPS) 2023*) (73) highlighted associations of treatment with exogenous insulin with weight gain and excess risk of cardiovascular outcomes (23, 24, 33, 74–76), similar to risks associated with sulfonylurea use. Treatment failure ensues quickly with sulfonylureas, as observed in clinical practice and in the large, recent GRADE Study (75). This treatment failure may be due to drug-related exhaustion of beta cells. Taken with the other risks of these agents, these two agents present less attractive benefits and risks, despite their ongoing widespread use. These authors encourage practitioners to weigh these disadvantages as they consider accessible options with more favorable benefit:risk profiles.

Hypoglycemia is a longstanding concern with choice of therapy. The risk of hypoglycemia increases with polypharmacy; polypharmacy is common in elderly patients treated in accordance with standards for preventative care and chronic disease management (77). The risks of severe hypoglycemic episodes are well known. It is less appreciated that recurrent, mild, asymptomatic hypoglycemia is also highly detrimental. In addition to counteracting efforts to normalize glucose, recurrent “nonsevere” hypoglycemic episodes incrementally contribute to cell damage (78), and the development of long-term complications. Large studies have shown that hyperglycemia, hypoglycemia (mild or severe), and glycemic variability are independently linked to the exacerbation of diabetes-related cognitive decline and dementia (67, 79). Moreover, each can increase the risk of onset of Alzheimer’s disease (25, 80, 81). Mild hypoglycemic episodes are difficult to detect, but newer tools such as continuous glucose monitoring have captured the frequency of these. Mild hypoglycemic excursions are particularly hard to avoid with use of sulfonylureas or insulin therapy (82). Nocturnal asymptomatic hypoglycemia occurs in approximately 25% of diabetic patients treated with insulin therapy, according to one report, and elderly patients are particularly susceptible (26). Patients with cognitive decline are another patient population who may be unaware of these fluctuations (26).

These authors have published on the associations between the dose of exogenous insulin and the risk of cardiovascular disease (23, 33). Large observational studies have shown dose-dependent associations for injected insulin with increased CV risk and worsened mortality, leading other workers to share concerns about use of insulin therapy and sulfonylureas in patients (31, 32, 83, 84).

Recent retrospective cohort studies among 132 737 insured adults with T2D revealed that compared with DPP-4 inhibitors, sulfonylureas were associated with an increased risk of cardiovascular events (HR, 1.36; 95% CI, 1.23-1.49). The risk associated with basal insulin was twofold greater (HR, 2.03; 95% CI, 1.81-2.27), while the CVD risk associated with other antidiabetic agents was similar to that associated with DPP-4 inhibitors (83). Another large analysis involving 15 studies revealed that, compared to patients who were not receiving insulin therapy, those who were receiving insulin therapy had a markedly increased risk of all-cause mortality (RR 1.46, 95% CI: 1.14, 1.88) and cardiovascular-specific mortality (RR 1.62, 95% CI: 1.33, 1.96) (84). Ke et al. reported a significant increase in the incidence of carotid plaque with insulin therapy compared to other therapeutic approaches (30). Insulin therapy was also associated with an increased risk of hospitalization and readmission (84–86). The health economic costs due to these outcomes is considerable.

A strong association between insulin secretagogues and the risk of all-cause mortality (MH-OR 1.11 [1.00, 1.23], p = 0.04) (32) was found in an analysis of 48 RCTs. According to another analysis, of 22 studies involving more than 200,000 participants, compared with basal insulin, GLP-1 receptor agonists had more favorable effects on composite cardiovascular outcomes (HR: 0.62, 95% CI: 0.48–0.79), heart failure (HR: 0.57, 95% CI: 0.35–0.92), myocardial infarction (HR: 0.70, 95% CI: 0.58–0.85), stroke (HR: 0.50, 95% CI: 0.40–0.63) and all-cause mortality (HR: 0.31, 95% CI: 0.20–0.48) (31). These studies have led to the guideline crafters of the *Obesity Medicine Association (OMA) Clinical Practice Statement 2023* to express concern for the cardiovascular safety risks with the use of exogenous insulin or sulfonylureas (73).

The risks of human insulin include weight gain, recurrent hypoglycemia (severe hypoglycemia as well as mild, ‘silent’ hypoglycemic episodes), and iatrogenic hyperinsulinemia, among other adverse effects. The results of overinsulinization due to use of injected insulin is also worth mention. Overinsulinization predisposes patients to inflammation, atherosclerosis, hypertension, dyslipidemia, heart failure (HF), and arrhythmias. Endogenous hyperinsulinemia is also suspected in CV and other problems (87).

## Application of metabolomics to T2D

The application of metabolomics has allowed for the broad discovery in the field of T2D - from biomarkers to etiological research. Poor glycemic control was found in one study to be correlated with alterations in 26 out of 162 metabolites common to metabolic regulation; predominant among these were glutamine and branched chain/aromatic amino acid changes (88). Another study revealed that specific metabolites were altered in diabetic youth compared with obese children without diabetes or healthy weight controls (89).

Research is also underway to capitalize on pharmacometabolomics as a means to identify biomarkers of treatment effectiveness, treatment decision-making tools, and new drug targets is ongoing. Metformin exerts a range of effects, some of which have been unexplained to date. Metabolomics has shown that metformin significantly impacts the levels of metabolites involved in the tricarboxylic acid cycle, the urea cycle, glucose metabolism, and lipid metabolism, emphasizing several pathways through which the broad effects of metformin are conferred (90).

Equally intriguing would be the discernment of the pathways through which GLP-1 receptor agonists exert protective effects on the cardiorenal axis. One recent study associated liraglutide with the metabolism of sphingolipids. Sphingolipids have been independently associated with the risk of atherosclerosis and cardiovascular disease (91). Sphingolipids provide structural support to cell membranes and influence an array of cell functions, including apoptosis and inflammation. This study not only resolved a pathway modulated by liraglutide, but may have identified a potential new drug target, sphingolipids.

## Genetics, inflammation and environmental factors

**Figure 3:**
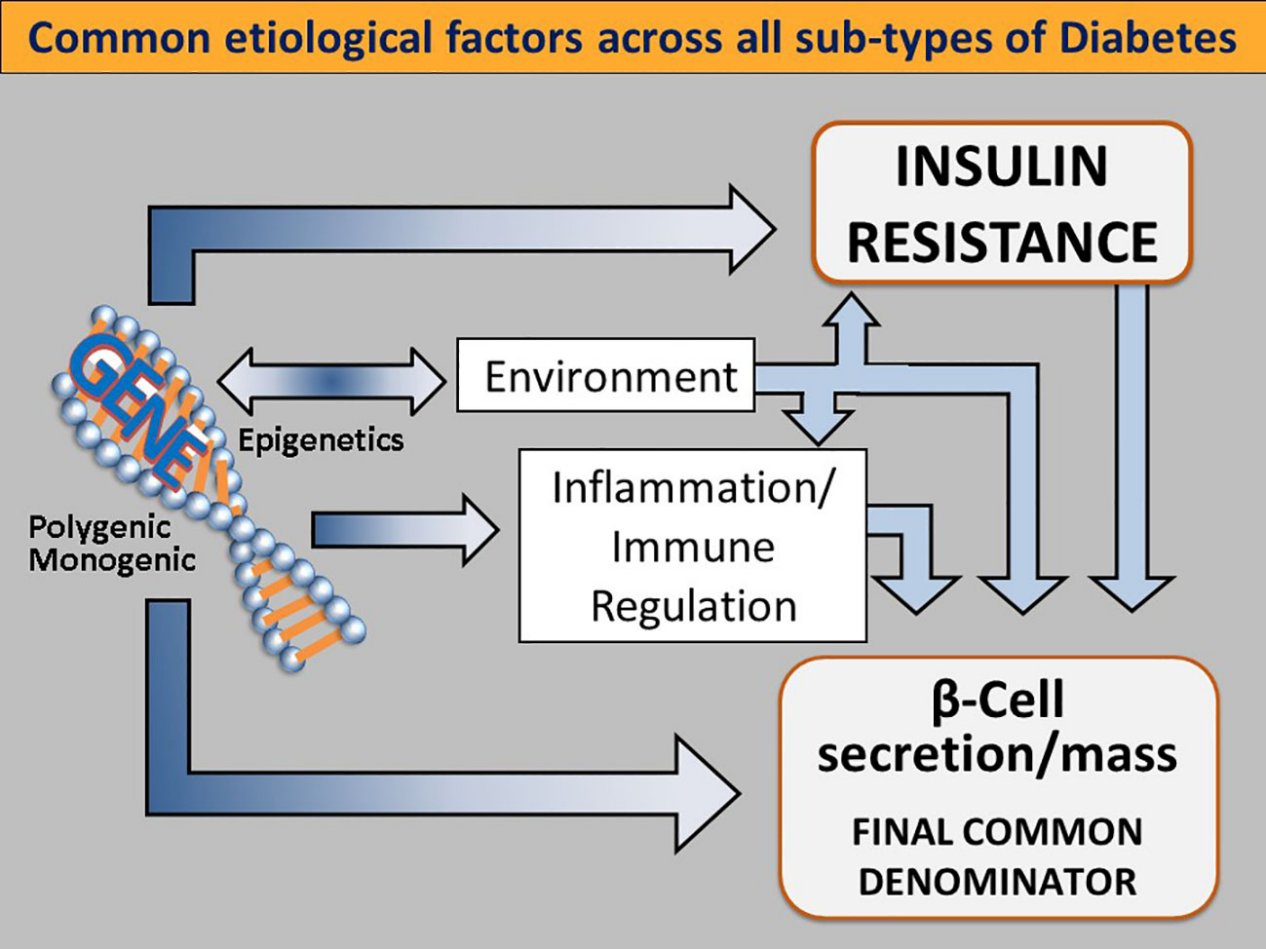
Common etiological factors across all subtypes of diabetes. Genetic determinants influence IR (either centrally or peripherally induced), loss of β-cell function and mass, environmental triggers (such as viruses, endocrine disruptors, advanced food glycosylation end products, and the gut microbiome), immune modulation and inflammation. Singly or more commonly, in various combinations, these factors converge on genetically susceptible β cells, impinge on β cell function and biology, and orchestrate the shift from normoglycemia to hyperglycemia. As this process takes place regardless of the subtype of DM, dysfunctional β-cells are the final common denominator in all DMs. Reused with permission.

A broad view of the diabetic state finds that there are also unmodifiable factors at work in dysglycemia. A portion of the development of T2D is subject to genetic, environmental and immune system factors. (**Figure 3**) These likely have evolved as adaptive metabolic survival strategies at times of nutrient unavailability or adverse environmental conditions (50–52).

A recent report in *Nature Genetics* characterized subpopulations of beta cells that were found to be involved in the progression from healthy cells to T2D (92). These cells responded to a transcriptional switch between HNF1A- and TCF4-defined gene regulatory programs. The regulatory programs influenced an array of processes, including insulin secretion, glucose transporters, suppressors of cytokine signaling, and ligand-gated calcium channels (92). This switch also orchestrated a core network of transcription factors and their target genes at the helm in determining beta cell subtypes (92–95).

Pancreatic function is linked to the immune system and influenced by macrophages and cytokines. Chronic inflammation has been shown to affect both the number and phenotype of macrophages, and negatively impact beta cell function and integrity (*reviewed by Serbis et al., 2023*) *(* 96). Ongoing research stands to identify not only the perturbations that cause beta cell dysfunction but also the innate protective factors within the immune system that could be therapeutically exploited.

The human gut microbiota contains ten times the number of human cells and exerts dramatic effects on metabolism and immune regulation. The gut microbiome is now understood to be a significant environmental influencer of T2D. Abnormal intestinal metabolites and intestinal barrier disruption are evident in T2D; and, can facilitate the entry of intestinal bacteria and their harmful metabolites into the circulatory system (3, 97). Moreover, there appears to be a bidirectional relationship between the gut microbiome and T2D, through which sustained hyperglycemia also influences the composition of the gut microbiome. This lends tantalizing possibilities that this is not a random, overlapping effect across two organ systems, but rather an evolutionary strategy to regulate organismal metabolism through the gut composition of bacteria. Fecal microbiota transplantation is being intensively studied, with compelling findings to date (98–100).

## Conclusions: T2D ‘2.0’

This review article poses that gold standards of T2D care have not been staying apace with our evolving knowledge of the disease. Past, erroneous beliefs about the etiology and progression of T2D must be rectified. An up-to-date functional knowledge of T2D is needed to provision practitioners to practice precision medicine and address the root cause(s) of T2D. New standards of optimal clinical care have been, and will continue to, supplant current standards of care.

The belief that overweight is the chief culprit in T2D needs to be revisited. In fact, excess nutrient intake is a greater contributor to the development of T2D (10). BMI measurements, however, miss the mark in that these tests do not estimate visceral fat, a more accurate predictor of T2D (11). This limits the reliability of BMI measurements for diagnostic purposes.

Hyperglycemia is only adequately managed in half of type 2 diabetes patients (101). A possible explanation is that the pharmacotherapeutics of choice are not fully ameliorating all of the pathophysiologies contributing to hyperglycemia in that patient. Under current best practices, when A1c targets prove illusory to achieve in the clinic, it is often assumed to be due to patient nonadherence. Alternately, it is estimated that 40% of hyperglycemia may be out of reach of our current interventions (1–3). Genetic, immune, and environmental contributors are also at work but are not modifiable by our antidiabetes armamentarium. The presence of unmodifiable factors should be considered risking tainting the trust between doctor–patient.

A major shift towards an accurate understanding of T2D is that the beta cells and glucoregulatory apparatus possess a resilience that can be leveraged until late in the disease. This has been a greatly underappreciated goal of therapy once pharmacotherapy is initiated. Remission is a realistic goal and should be considered in candidate patients from early stage disease — until the gluco-regulatory apparatus has been irreparably damaged by years of glucolipotoxicity. Regimens are established for use as short-term, intensive approaches that are evidenced to reset gluco-stasis and restore normoglycemia in many patients.

In the past, the choice of pharmacotherapy was based on the extent of A1c elevations. Uncontrolled plasma glucose prompted add-on therapy without regard to the individual drivers of hyperglycemia at work in the patient. As a corollary to this, any therapies that do not target one of the defects present in the patient can be discontinued. Patient comorbid conditions are now major considerations within treatment decision trees.

As our antidiabetes armamentarium expands, we exact intensive glucose control with less risk of hypoglycemia. We are now cognizant of the true harm of hypoglycemia, including ‘silent’ hypoglycemia excursions and glucose variations. Glucose variation even in the absence of severe hypoglycemic episodes is associated with poorer long-term outcomes.

We have emphasized the essential role of beta cells in maintaining and restoring normoglycemia. The functional integrity of these precious beta cells has not been targeted by any of our long list of current antidiabetes classes. Diminished mitochondrial function has been identified as a key defect in beta cell failure and insulin resistance (102, 103). A new agent, imeglimin, targets defective cellular energy metabolism in T2D (104, 105), presumably by protecting beta cells from oxidative stress and endoplasmic reticulum stress (104, 105). Imeglimin exhibits a generally favorable safety and tolerability profile, including lack of severe hypoglycemia (106). Imeglimin was commercialized in Japan in 2021, and, may be launched worldwide. Other treatments in development that may improve beta cell function include calcium channel blockers, which may protect against beta cell apoptosis (107, 108), and verapamil, which may help to preserve beta cell function in T1D patients (109).

Perhaps the holy grail of T2D treatment will be beta cell neogenesis as a therapeutic target to repopulate damaged or depleted beta cells (110). Approaches in in early development for the treatment of T2D span antibodies, receptor antagonists, natural compounds and small molecules (28). Perhaps one or more of these will fill this gap. In the meantime, protection of the beta cells it is paramount in clinical management. Some currently available therapies may support – or do no or little to decrease the the longevity of beta cells, while other approaches, namely, sulfonylureas or human insulin, may further tax beta cells, accelerating their exhaustion.

## Author contributions

SS: Conceptualization, Writing – review & editing. MH: Conceptualization, Writing – original draft, Writing – review & editing.
